# The safety of remimazolam versus propofol in gastroscopic sedation: a meta-analysis

**DOI:** 10.1186/s12871-024-02422-y

**Published:** 2024-01-29

**Authors:** Xincan An, Tianqi Shen, Xingxing Yin, Jin Xu, Yongming Zhang, Tianlong Wang

**Affiliations:** 1 Department of Anesthesiology, Xuanwu Hospital, Capital Medical University, Beijing, China; 2 Department of Anesthesiology, 984th Hospital of the People’s Liberation Army, Beijing, China; 3 Department of Education, Xuanwu Hospital, Capital Medical University, Beijing, China

**Keywords:** Remimazolam, Propofol, Gastroscopic sedation, Meta-analysis

## Abstract

**Background:**

This meta-analysis was designed to compare the safety and efficiency of remimazolam with those of propofol in patients undergoing gastroscope sedation.

**Methods:**

We searched PubMed, Cochrane Library, Embase, Ovid, Wanfang Database, China National Knowledge Infrastructure, SINOMED, and ClinicalTrials.gov for studies that reported on remimazolam versus propofol for gastroscope sedation from establishment to February 25, 2023. The sedative efficiency and the incidence of adverse events were assessed as outcomes. Version 2 of the Cochrane risk-of-bias assessment tool was used to assess the risk of bias. Review Manager 5.4 and STATA 17 were used to perform all statistical analyses.

**Results:**

A total of 26 randomized controlled trials involving 3,641 patients were included in this meta-analysis. The results showed that remimazolam had a significantly lower incidence of respiratory depression (risk ratio [*RR*] = 0.40, 95% confidence interval [CI]: 0.28–0.57; *p* < *0.01*, GRADE high), hypoxemia (*RR* = 0.34, 95% CI: 0.23–0.49; *p* < *0.01*, GRADE high), bradycardia (*RR* = 0.34, 95% CI: 0.23–0.51; *p* < *0.01*, GRADE high), dizziness (*RR* = 0.45, 95% CI: 0.31–0.65; *p* < *0.01*, GRADE high), injection site pain (*RR* = 0.06, 95% CI: 0.03–0.13; *p* < *0.01*, GRADE high), nausea or vomiting (*RR* = 0.79, 95% CI: 0.62–1.00; *p* = 0.05, GRADE moderate), and hypotension (*RR* = 0.36, 95% CI: 0.26–0.48; *p* < *0.01*, GRADE low).

**Conclusions:**

Remimazolam can be used safely in gastroscopic sedation and reduces the incidence of respiratory depression, hypoxemia, bradycardia, injection site pain, and dizziness compared with propofol, and doesn't increase the incidence of nausea and vomiting.

**Supplementary Information:**

The online version contains supplementary material available at 10.1186/s12871-024-02422-y.

## Introduction

Gastroscopy is the gold standard evaluation modality for upper gastrointestinal tract diseases [1]. However, gastroscopy without any anesthesia causes discomfort in patients. Gastroscopy after the administration of general anesthesia by the anesthesiologist improved the patient’s comfort and satisfaction, decreased stress response and the occurrence of adverse reactions, and elevated security [2, 3].

Currently, one of the most widely used sedative agents is propofol [4]—a short-acting anesthetic commonly used in clinical settings. It has the advantages of rapid onset of action and recovery and no obvious accumulation after long-term infusion. It is widely used in anesthesia induction, anesthesia maintenance of various surgeries, and painless endoscopy. Nonetheless, it has been reported that propofol can cause respiratory and cardiovascular depression, hypoxia, and injection site pain [5].

Remimazolam is a new short-acting benzodiazepine that acts as a γ-aminobutyric acid subtype A (GABA-A) receptor agonist [6]. Its properties include rapid onset, a short duration of sedation, and full recovery. Remimazolam is characterized by a metabolism mechanism independent of liver and kidney function and is rapidly metabolized to inactive compounds by tissue esterases [7]. A phase III trial involving adult patients showed that remimazolam has a lower incidence of hypotension than propofol [8]. Additionally, the pharmacological effects of remimazolam can be quickly reversed by the specific antagonist flumazenil, which provides a relatively safer profile.

Studies have found that remimazolam has a sedative effect similar to that of propofol. They have similar success rates of sedation and depth of sedation [9, 10] but remimazolam has a lower incidence of respiratory and circulatory inhibition. However, the induction time, incidence of postoperative nausea and vomiting(PONV), and other outcomes have diametrically opposite results. Considering the different sedation needs and anesthetic concerns of different endoscopic examinations, we only included studies on the use of remimazolam in gastroscopy, while focusing on outcome indicators of airway protection, such as respiratory depression, hypoxemia, and coughing, to explore the differences in the effects between remimazolam and propofol.

## Material and methods

The protocol of this systematic review follows the Preferred Reporting Items for Systematic Reviews and Meta-Analyses (PRISMA) 2020 statement [11] and was registered at PROSPERO (ID: CRD42023399639; Date: Feb 25, 2023).

### Data sources and search strategy

All authors independently searched studies from PubMed, Cochrane Library, Embase, Web of Science (WOS) Core database, Ovid, Wanfang Database, China National Knowledge Infrastructure, and SINOMED from establishment to February 25, 2023. We also searched the American Anesthesia Association website and other websites for unpublished research data. We used “remimazolam”, “gastroscope”, “propofol” and their synonyms for subject and free word searches. We manually searched the references of the retrieved studies as a supplement to avoid missing relevant documents. For example, the retrieval formula in PubMed is shown in Table S1.

### Inclusion and exclusion criteria

The inclusion criteria were as follows: (1) the experimental design was a randomized controlled trial (RCT); (2) the research objects were patients undergoing painless gastroscopy who needed anesthesia, regardless of sex or nationality; and (3) remimazolam was used as the only sedative in the test group, and propofol was the only sedative in the control group, with or without one or more opioid analgesics.

The exclusive criteria were as follows: (1) the outcome does not include the following: ① primary outcomes: the incidence of any adverse events, including respiratory depression, hypoxemia, hypotension, bradycardia, cough, nausea and vomiting, dizziness, body movement, and injection site pain, ② secondary outcomes: induction time, recovery time, discharge time; (2) phase I or II clinical trials; (3) different opioid analgesics were administered in two groups; and (4) no exact induction dose of remimazolam per body weight.

### Literature perusing and data extraction

The review authors independently screened the titles and abstracts of the studies identified by our search and excluded obviously irrelevant studies according to the inclusion and exclusion criteria. The remaining studies were read in-depth, and the authors selected the required studies according to the inclusion and exclusion criteria. The review authors independently extracted data from the included studies, as follows: name of the first author, year of study, general patient information (e.g., age, body mass index, and American Society of Anesthesiologists classification), dose of remimazolam, intraoperative analgesics, and outcomes. For multi-arm studies, we selected data from the control and experimental groups recommended by the final results of the study to be included in this review [12].

### Quality and bias evaluation

All authors independently assessed the methodological quality of the included studies. The risk of bias was assessed using version 2 of the Cochrane risk-of-bias assessment tool [13], including bias arising from the randomization process, bias due to deviations from intended interventions, bias due to missing outcome data, bias in measurement of the outcome, bias in selection of the reported result, and overall risk of bias. If the other authors were at odds, the corresponding author decided the bias.

### Data processing and analysis

Review Manager 5.4 and STATA 17 were used to analyze the results. Continuous outcomes were represented by mean differences (*MDs*) and 95% confidence intervals (95%CI), and dichotomous outcomes were represented by risk ratios (*RRs*) and 95% CIs. For continuous variable data that only report medians and interquartile ranges (*IQR*), we managed the data following the advice in the Cochrane Handbook for Systematic Reviews of Interventions [14]. Pooled effects were computed using inverse-variance weighted effects. Heterogeneity analysis was performed among the included studies. A value of *I*^2^ > 50% indicated significant heterogeneity, where a random-effects model was used. Otherwise, a fixed-effects model was used if *I*^2^ ≤ 50% [15]. We also searched for the source of heterogeneity using subgroup analysis. The Harbord and Egger tests were used to test for publication bias, and sensitivity analysis was performed to assess whether the final results were stable.

### Grading of evidence

We rated the evidence obtained in terms of the risk of bias, inconsistency, non-directness, imprecision, and other aspects, as recommended by the Grading of Recommendations, Assessment, Development, and Evaluation guidelines [16–18]. We calculated the optimal information size [19] for each outcome indicator (assuming α of 0.05 and β of 0.1). We used the GRADEpro GDT online software to assess the certainty of the evidence and to create a summary table of the results.

## Results

### Search results

**Fig. 1:**
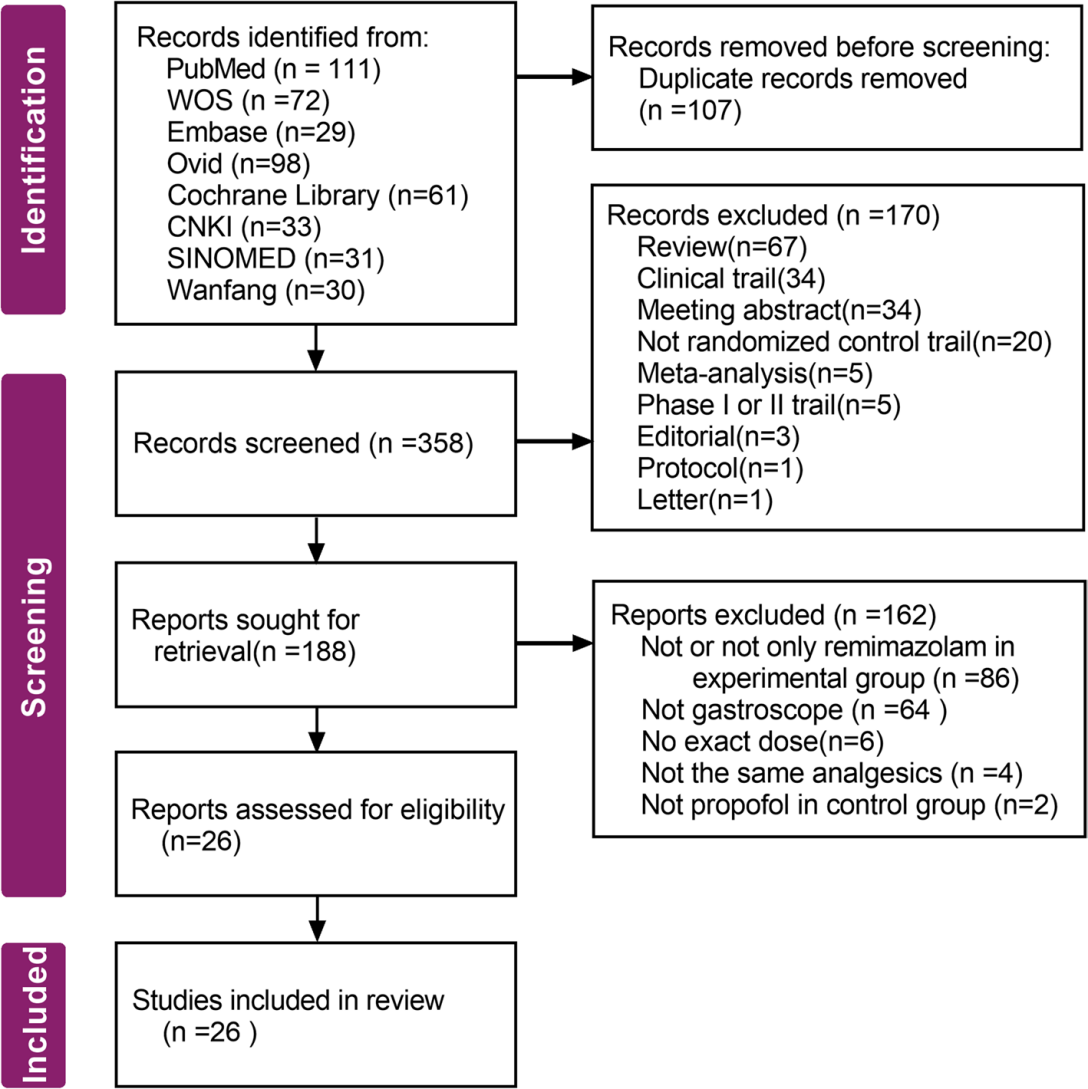
Identification of studies via databases and registers

In this study, 465 articles were identified by keyword search. According to the inclusion and exclusion criteria, we reduced the number to 26 trials [20–45] with 3,641 patients for this meta-analysis (Fig. 1). The main characteristics of the 26 RCTs are summarized in Table S2. All studies were conducted in China, of which 20 RCTs were published in China. Nine RCTs [21, 24, 25, 29, 30, 32, 38, 42, 45] involved old individuals aged more than 60 years, and one RCT [23] involved children aged less than 18 years.

### Meta-analysis results

#### Meta-analysis (Fig. 2)

**Fig. 2:**
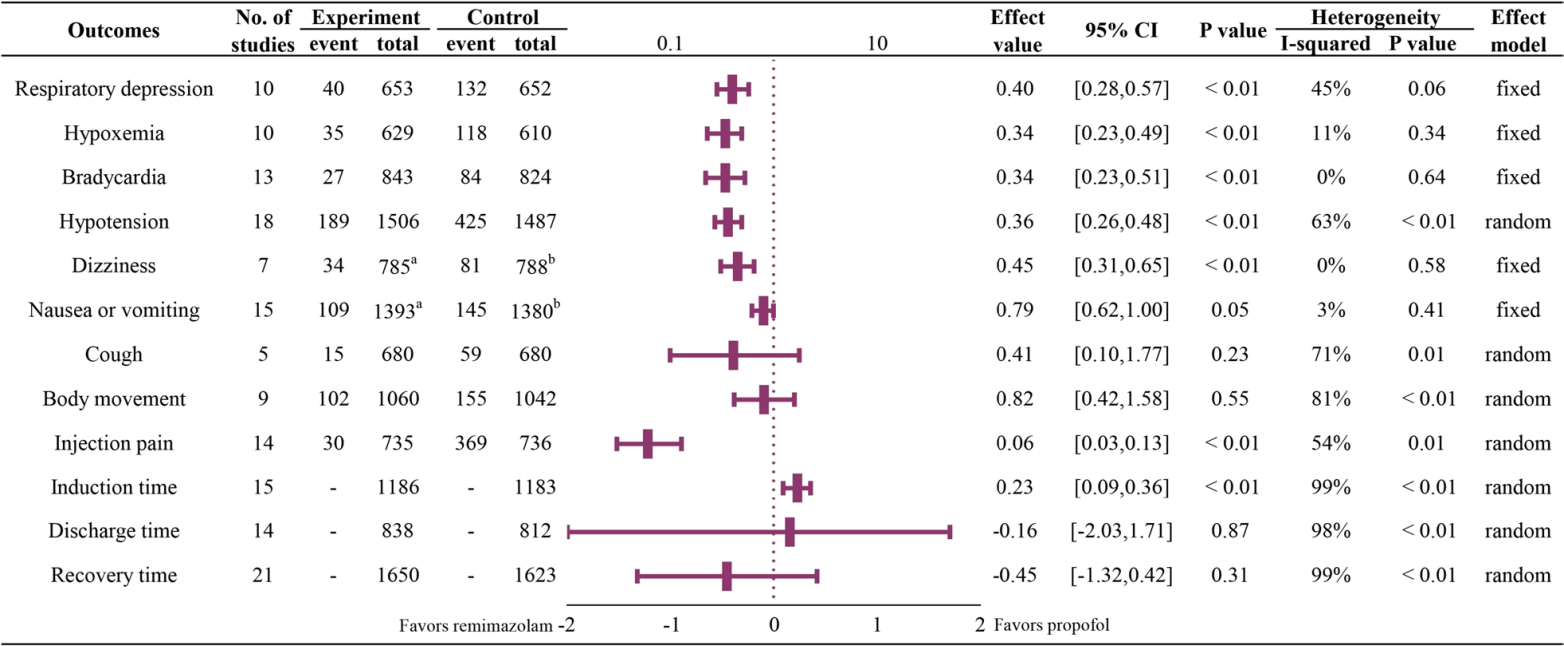
Summary of forest plots for all outcomes; a. 6 cases missing; b. 1 case missing

For the respiratory system, 10 RCTs [21, 27, 30, 32, 33, 35, 36, 42–44] with 1,305 patients reported the incidence of respiratory depression. The incidence of respiratory depression was significantly lower in the remimazolam group than in the propofol group (*RR* = 0.40, 95% CI: 0.28–0.57; *p* < 0.01), with lower heterogeneity (*I*^*2*^ = 45%, *p* = 0.06) (Figure S1). Ten studies [22–24, 28, 29, 31, 32, 34, 38, 41] involving 1,239 patients reported the incidence of hypoxemia; the incidence of hypoxemia was significantly lower in the remimazolam group (*RR* = 0.34, 95% CI: 0.23–0.49; *p* < 0.01), with lower heterogeneity (*I*^2^ = 11%, *p* = 0.34) (Figure S2).

For the circulatory system, 13 studies [22–24, 27–29, 31, 32, 34–36, 43, 45], involving 1,667 patients, assessed the incidence of bradycardia. The incidence of bradycardia was significantly lower in the remimazolam group (*RR* = 0.34, 95% CI: 0.23–0.51; *p* < 0.01) (*I*^2^ = 0%, *p* = 0.64) (Figure S3). Furthermore, 18 studies [22–24, 27–32, 34–36, 38–40, 42, 43, 45], involving 2,993 patients, assessed the incidence of hypotension. The incidence of hypotension was lower in the remimazolam group than in the propofol group (*RR* = 0.36, 95% CI: 0.26–0.48; *p* < 0.01) with significant heterogeneity (*I*^2^ = 63%, *p* < 0.01) (Figure S4).

The incidence of dizziness in the remimazolam group was less than that in the propofol group. Seven studies [21, 22, 24, 27, 29, 38, 40] assessed the incidence of dizziness in 1,573 patients and showed a statistically significant difference (*RR* = 0.45, 95% CI: 0.31–0.65; *p* < 0.01) between the two groups with low heterogeneity (*I*^2^ = 0%, *p* = 0.58) (Figure S5). Furthermore, 15 studies [21, 22, 24, 27, 32–38, 40, 42, 43, 45], involving 2,773 patients, reported the incidence of nausea and vomiting. No significant difference was observed between the two groups (*RR* = 0.79, 95% CI: 0.62–1.00; *p* = 0.05) (*I*^2^ = 3%, *p* = 0.41) (Figure S6).

No significant difference in the incidence of cough [28, 38, 40, 42, 44] and body movement [27, 28, 32, 34, 36, 38, 40–42] was observed between the remimazolam and propofol groups (*RR* = 0.41, 95% CI: 0.10–1.77, *p* = 0.23; *RR* = 0.82, 95% CI: 0.42–1.58, *p* = 0.55). However, the incidence of both outcomes showed a significant difference between studies (*I*^2^ = 71%, *p* = 0.01; *I*^2^ = 81%, *p* < 0.01) (Figure S7 and Figure S8).

Fourteen studies [21–23, 29, 30, 32, 33, 35–38, 41, 43, 44], involving 1,471 patients, demonstrated the occurrence of injection site pain. The incidence of injection site pain was significantly lower in the remimazolam group than in the propofol group (*RR* = 0.06, 95% CI: 0.03–0.13; *p* < *0.01*), with high heterogeneity (*I*^2^ = 54%, *p* = 0.01) (Figure S9).

For time correlation outcomes, remimazolam takes a longer time to induce sedation [21–23, 25–30, 32, 33, 36, 37, 40, 43] (*MD* = 0.23 min, 95% *CI*: 0.09 to 0.36 min; *p* < 0.01), but similar time for discharge [20–22, 24, 25, 27, 28, 30, 32–34, 36–44](*MD* =  − 0.16 min, 95% *CI*: − 2.03 to 1.71 min; *p* = 0.87) and recovery time [20, 22, 24, 28–30, 32–37, 41, 44, 45] (*MD* = -0.45 min, 95% *CI*: − 1.32 to 0.42 min; *p* = 0.31). All of them showed significant heterogeneity between studies (*I*^2^ = 99%, *p* < 0.01; *I*^2^ = 98%, *p* < 0.01; *I*^2^ = 99%, *p* < 0.01) (Figure S10; Figure S11; Figure S12).

#### Subgroup analysis and meta regression

We performed a subgroup analysis of the results with significant heterogeneity and included more than ten studies on different doses. The doses of remimazolam in the included studies ranged from 0.1–0.55 mg/kg, with a median of 0.2 mg/kg. Thus our dose subgroup was based on the reported dose of remimazolam per body weight, interpreted as follows: ≤ 0.15 mg/kg as low, ≥ 0.25 mg/kg as high, and the rest as medium. Simultaneously, we performed meta-regression on the logarithm of RR and the dose of remimazolam.

**Fig. 3:**
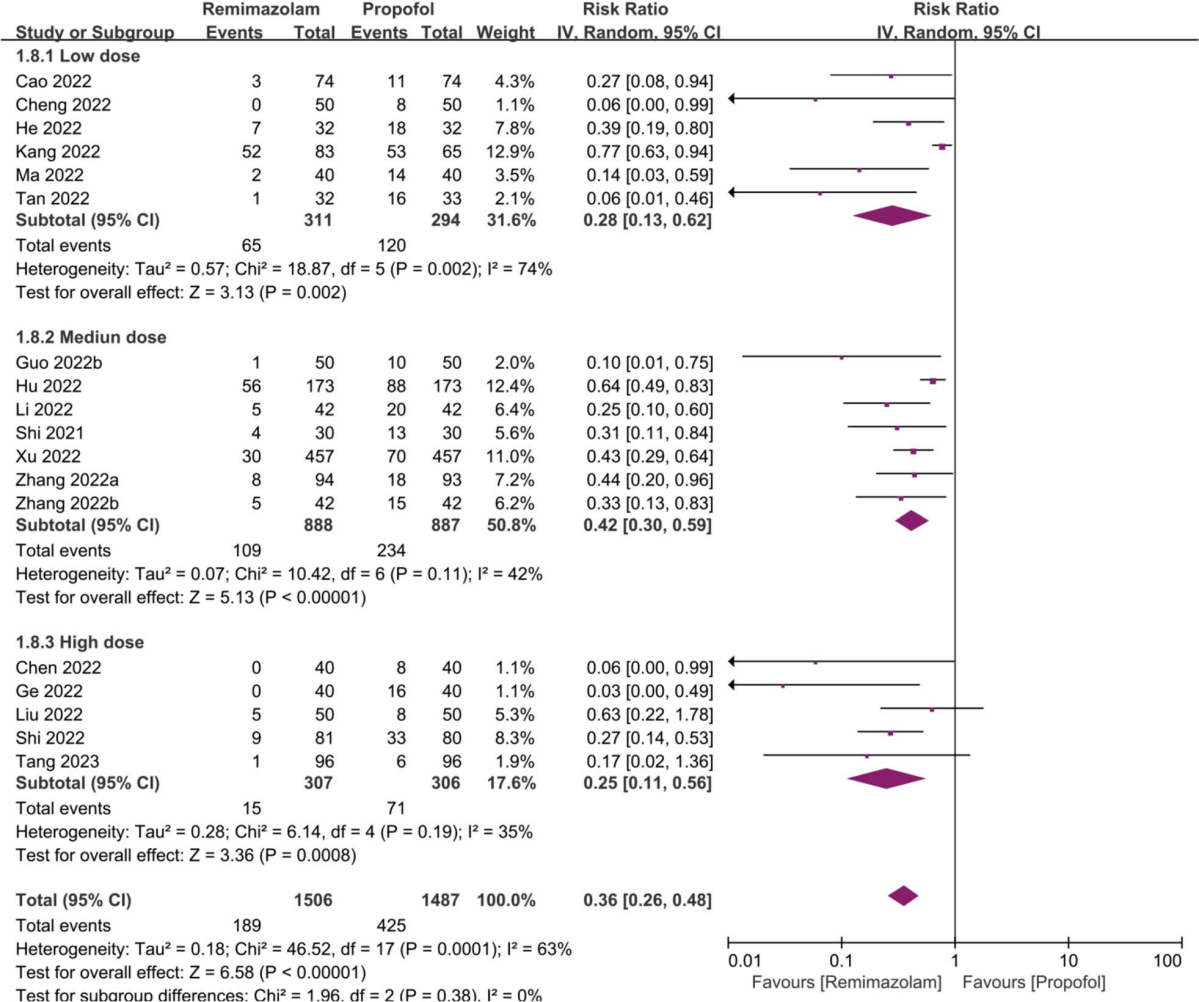
Forest plots of Hypotension by dose

In the subgroup analysis of hypotension, low within-group heterogeneity was observed in medium dose and high dose subgroups (*I*^2^ = 42%, *p* = 0.11; *I*^2^ = 35%, *p* = 0.19), but high heterogeneity was found in low dose subgroup (*I*^2^ = 74%, *p* < 0.01). However, there was no significant difference between subgroups (*P* = 0.38). All subgroup results favored remimazolam (Fig. 3).

**Fig. 4:**
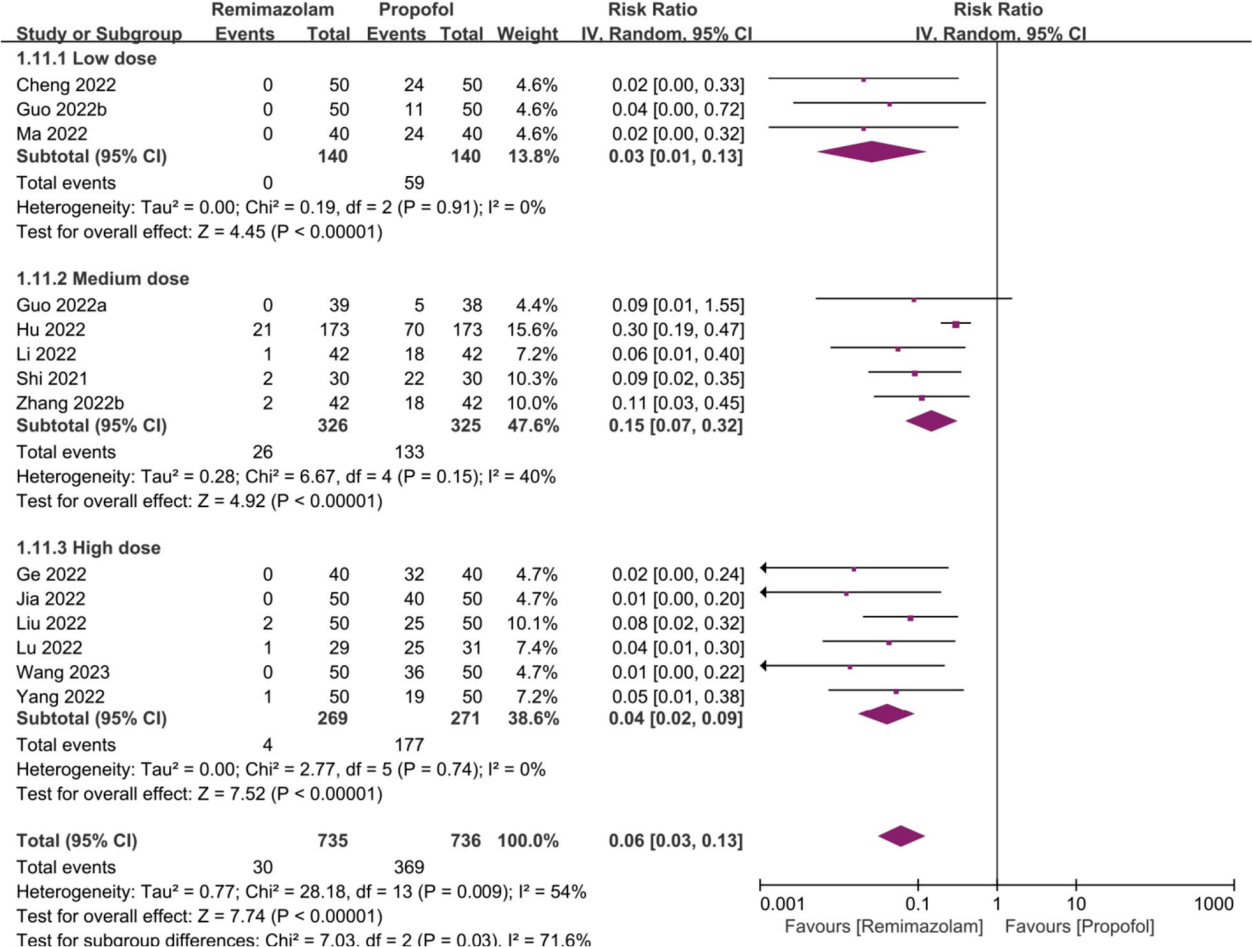
Forest plots of Injection pain by dose

For injection site pain, all three dose subgroups showed no statistically significant heterogeneity within subgroups(*I*^2^ = 0%, *p* = 0.91; *I*^2^ = 40%, *p* = 0.15; *I*^2^ = 0%, *p* = 0.74), and all subgroup results favored remimazolam (Fig. 4).

Immediate dose was an influential factor in the heterogeneity of injection site pain, however, meta-regression showed that this did not fit linear regression (*P* = 0.478, Ajd *R*^2^ = 2.31%). All studies reporting injection pain did not use remimazolam besylate, Including the study [32] that presented different results in sensitivity analysis.

In terms of time-related outcome indicators, all subgroups showed high heterogeneity. The effect values of each outcome indicator were so close to or crossed the null line that they exhibited some instability in the subgroup analysis.

#### Sensitivity analysis

**Fig. 5:**
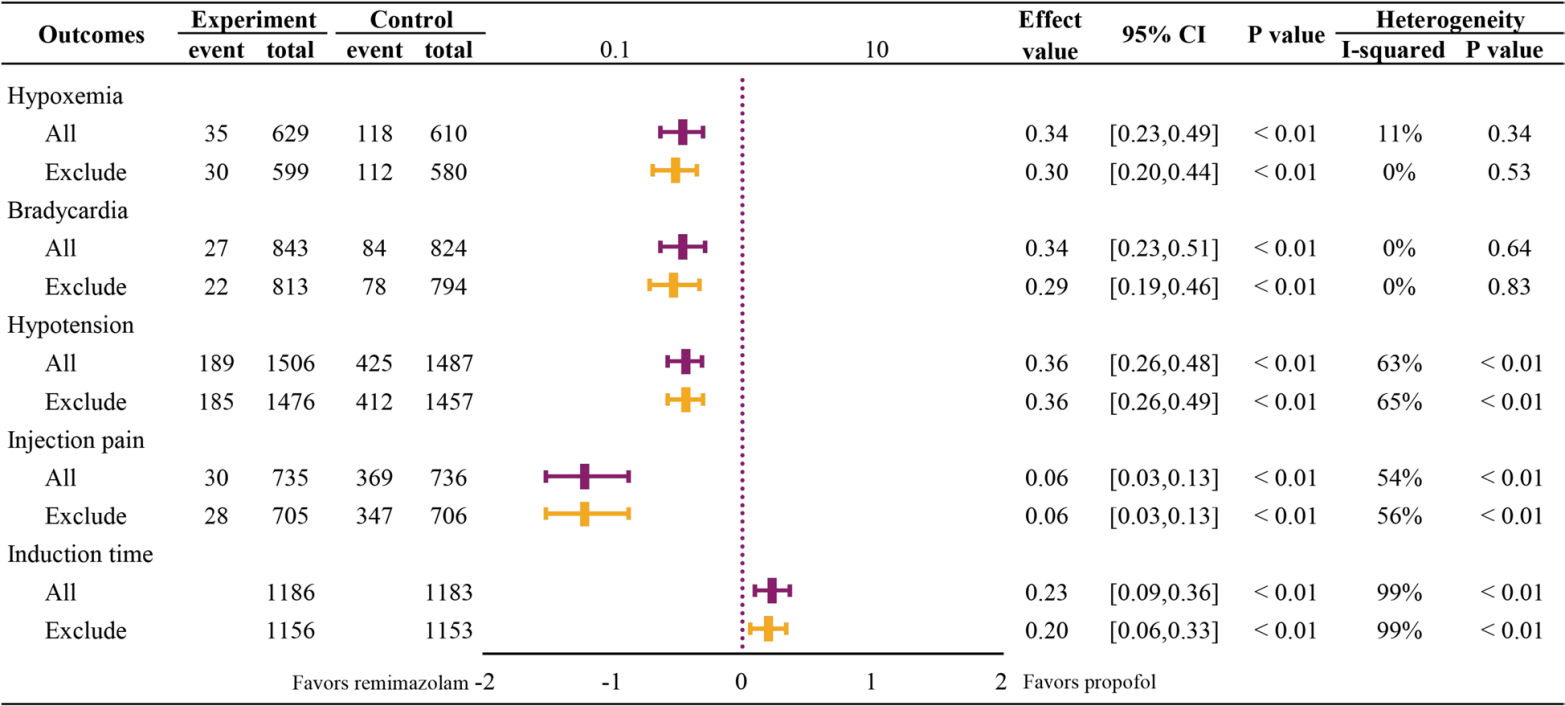
Sensitivity analysis for the only pediatric study

Considering that only one of the included studies involved children and the average age was less than three years old, we conducted a sensitivity analysis for this study. It was found that the exclusion of this study did not affect the results or the heterogeneity (Fig. 5).

We used the one by one elimination method to analyze the sensitivity of the results when included in no less than 10 studies. Except for Nausea or vomiting, recovery time, and discharge time, all results have good stability. However, no possible sources of heterogeneity were found in the studies that produced the opposite results (Figure S13).

For injection pain, the change in heterogeneity after eliminated one study [32] became low (Figure S14). Consider this study as a source of heterogeneity, but no possible reason was found in the study.

### Risk of bias assessment

#### Bias assessment

**Fig. 6:**
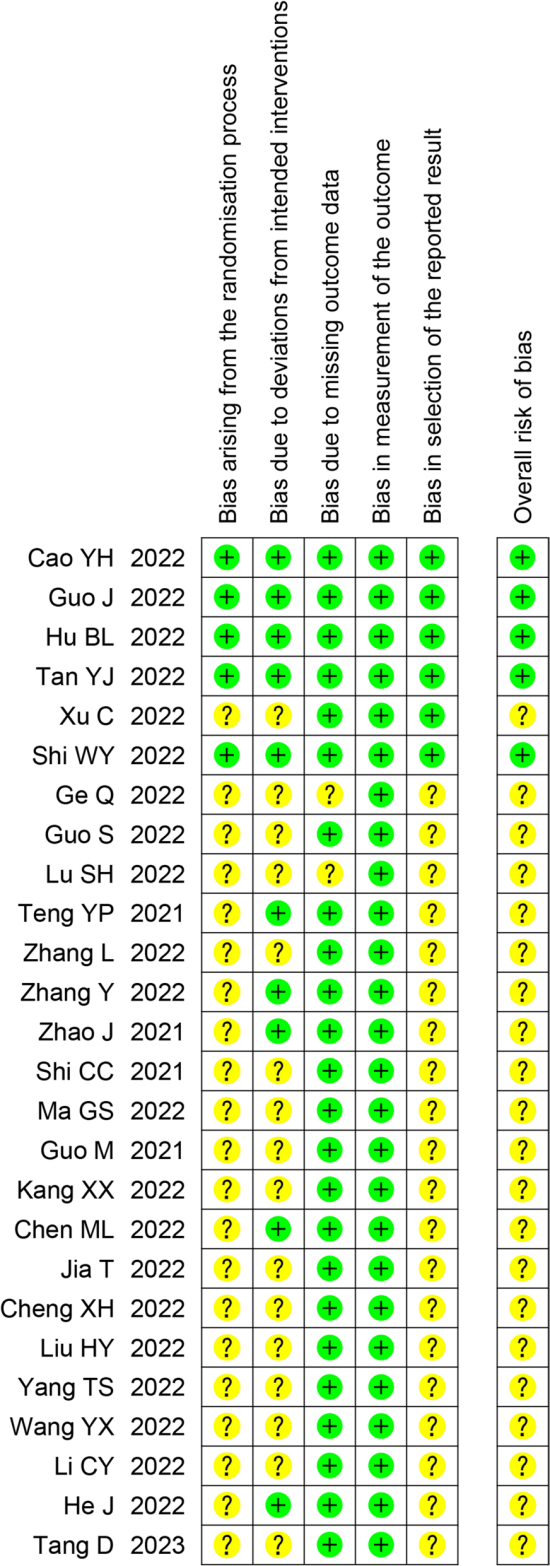
Risk of bias summary

All studies adopted a randomization method; however, three of them did not clearly describe the method. Blinding was explicitly mentioned in seven studies. Only six [21, 24, 27, 32, 39, 40] studies have been registered, and task plans could be queried. All studies reported the outcomes completely, but only five studies assessed low risk in overall results. (Fig. 6 and Figure S15).

### Report bias

**Fig. 7:**
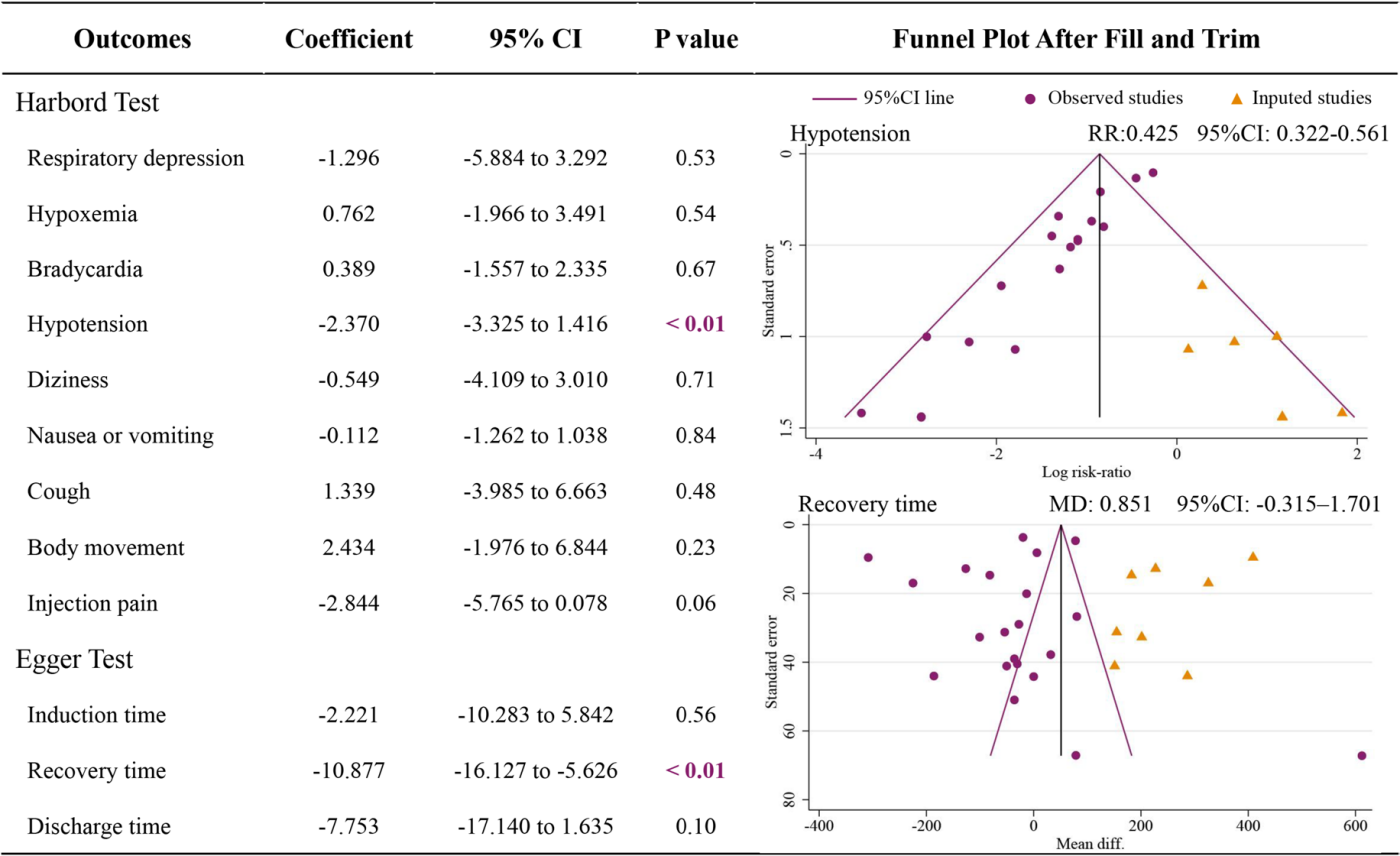
Results of repot bias and trim & fill method

We performed the Harbord test for the binary indicators and the Egger test for the continuous variable indicators [46]. The results showed that there is report bias in hypotension (*p* < 0.01) and recovery time (*p* < 0.01). We used the trim and filling method to adjust the results. After three iterations, six studies were filled with the result of the incidence of hypotension. The result after filling stayed similar (*RR* = 0.425, 95% CI: 0.322 – 0.561). The recovery time was still not statistically significant after five iterations and filling eight studies (*MD* = 0.851 min, 95% *CI*: -0.315 to 1.701 min) (Fig. 7).

### Summary of evidence

We used the GRADEpro GDT online software to grade the evidence for 7 primary outcomes covering more than 10 studies. Regarding the risk of bias, all outcomes were downgraded as serious as the overall bias was medium risk. Two outcomes were graded serious in terms of consistency because *I*^2^ > 50%, and one outcome was downgraded for detected publication bias. Six were upgraded due to strong association, one of which was classified as very strong association. Therefore, five pieces of evidence were classified as high, one as moderate, and one as low (Table 1).

**Table 1:** Summary of findings

Certainty assessment						No. of patients		Effect		Certainty
**No. of studies**	**Risk of bias**	**Inconsistency**	**Indirectness**	**Imprecision**	**Other considerations**	**remimazolam**	**propofol**	**Relative (95% CI)**	**Absolute (95% CI)**	
**Respiratory depression**										
10 RCTs	**serious**^a^	not serious	not serious	not serious	**strong association**	40/653 (6.1%)	132/652 (20.2%)	**RR 0.40** (0.28 to 0.57)	121 fewer per 1,000 (from 146 to 87 fewer)	⊕ ⊕ ⊕ ⊕ **High**
**Hypoxemia**										
10 RCTs	**serious**^a^	not serious	not serious	not serious	**strong association**	35/629 (5.6%)	118/610 (19.3%)	**RR 0.34** (0.23 to 0.49)	128 fewer per 1,000 (from 149 to 99 fewer)	⊕ ⊕ ⊕ ⊕ **High**
**Bradycardia**										
13 RCTs	**serious**^a^	not serious	not serious	not serious	**strong association**	27/843 (3.2%)	84/824 (10.2%)	**RR 0.34** (0.23 to 0.51)	67 fewer per 1,000 (from 78 to 50 fewer)	⊕ ⊕ ⊕ ⊕ **High**
**Dizziness**										
7 RCTs	**serious**^a^	not serious	not serious	not serious	**strong association**	34/785 (4.3%)	81/788 (10.3%)	**RR 0.45** (0.31 to 0.65)	57 fewer per 1,000 (from 71 to 36 fewer)	⊕ ⊕ ⊕ ⊕ **High**
**Injection pain**										
14 RCTs	**serious**^a^	**serious**^b^	not serious	not serious	**very strong association**	30/735 (4.1%)	369/736 (50.1%)	**RR 0.06** (0.03 to 0.13)	471 fewer per 1,000 (from 486 to 436 fewer)	⊕ ⊕ ⊕ ⊕ **High**
**Nausea or vomiting**										
15 RCTs	**serious**^a^	not serious	not serious	not serious	none	109/1393 (7.8%)	145/1380 (10.5%)	**RR 0.79** (0.62 to 1.00)	22 fewer per 1,000 (from 40 to 0 fewer)	⊕ ⊕ ⊕ ⊝ **Moderate**
**Hypotension**										
18 RCTs	**serious**^a^	**serious**^b^	not serious	not serious	**publication bias strongly suspected** ^c^; **strong association**	189/1506 (12.5%)	425/1487 (28.6%)	**RR 0.36** (0.26 to 0.48)	183 fewer per 1,000 (from 211 to 149 fewer)	⊕ ⊕ ⊝ ⊝ **Low**

**Explanations**

^a^Most information is from studies at moderate risk of bias

^b^High heterogeneity(I-squard > 50%)

^c^Publication bias detected by Harbord test

## Discussion

Sedation and anesthesia in gastroscopy have become an indispensable medical treatment, which can not only give patients a comfortable medical experience, but also facilitate the operator to perform the examinations, and improve safety by inhibiting unexpected body movements or coughing reactions [47]. However, different anesthetics may bring different sedative effects and adverse reactions, such as hemodynamic instability, respiratory depression, and so on [48].

This study has shown that the incidence of hypotension and bradycardia in the remimazolam group was significantly lower than that in the propofol group. Although hypotension had high heterogeneity and publication bias, the results after sensitivity analysis or the trim and filling method remained stable. Dogan’s research has found that contrary to the compensatory increase in the sympathetic dominance of propofol, remimazolam has not changed the balance between sympathetic and parasympathetic activities, reducing the fluctuation in the circulatory system [49]. Studies have shown that remimazolam mechanism that regulates the bradykinin B_1_ receptor and autophagy to relieve the pain [50], which may benefit circulation stability.

The incidence of respiratory depression and hypoxemia also favored remimazolam with a higher evidence grade. Research on the mechanism of respiratory inhibition of propofol by Jiang showed that propofol may bind to β_3_, which mediates respiratory depression and loss of consciousness [51], whereas the four receptor subtypes to which remimazolam binds are associated the β_2_ subunit [7], which may explain the incidence of less respiratory function inhibition during the application of remimazolam; however, the differences between different GABA subtypes need more in-depth research.

This meta-analysis showed that the incidence of injection site pain during gastroscopy can be reduced when using remimazolam. The results were stable despite having high heterogeneity. Similar findings have been reported in relevant studies outside this study [52]. The incidence of injection site pain induced with propofol has been reported to be > 66% [53]. Lidocaine, as an effective adjuvant known to inhibit injection site pain [54], still results in injection site pain in 30% of patients [55] and even the hardest part of the anesthesia process for some patients [56]. Pain by injection may be linked to the stimulation of lipid components in the compatibility of propofol on blood vessels. The difference is that remimazolam is water-soluble; therefore, it has less tissue stimulation and less incidence of pain by injection.

This study showed no statistically significant difference between propofol and remimazolam in the incidence of PONV, and both had an incidence of less than 20%, which is lower than the 25%-50% incidence reported in the literature [57]. Using opioid analgesics in gastroscopy is a risk factor for early PONV. In this study, in order to minimize differences due to opioids, we excluded trials with different opioid analgesics or different doses between the two groups. Most of the studies that were included used alfentanil and sufentanil. However, the effect of different opioids on the incidence of PONV needs to be further investigated. Although a previously published meta-analysis has shown that midazolam reduces the incidence of PONV compared to placebo [58], but the mechanism of action is unclear. Propofol has been widely demonstrated to be effective in reducing the incidence of PONV [59] but is not evident during subhypnotic infusion [60]. Compared with general anesthesia surgery, gastroscopy examination is shorter and fewer anesthetic drugs are injected during anesthetic sedation, which may weaken the inhibitory effect of propofol on PONV. This may explain why this meta-analysis showed that remimazolam is similar to propofol in terms of incidence of nausea and vomiting after gastroscopy, which is similar to the previously published meta-analysis results of midazolam [61], but further research is needed to determine whether remimazolam has the same effect as propofol in reducing PONV.

Cough and body movement did not show a difference between the two groups in this study, as there are many factors that can influence this, such as dose, age, criteria, analgesic medication, and even operator technique. Using both cough and body movement as criteria and outcome indicators may add bias to the experimental design. The spectral edge frequency (SEF) can be closer to the depth of sedation of remimazolam than the bispectral index and MOAA/S [62, 63]. The application of the SEF in gastroscopic sedation may bring higher evidence for cough and body movement.

For the time-related outcomes, only the induction time was slightly slower in remimazolam group than propofol, but the SMD between the two groups was not sufficient to produce a clinically significant difference. Heterogeneity was high for all three outcomes, but no potential confounding factors were identified that might be related to the method of outcome measurement.

This study confirms that remimazolam has advantages over propofol in terms of cardiopulmonary depression and injection site pain, as well as a lower risk of abuse [64] compared to the potentially addictive properties of propofol [65]. However, as a member of the benzodiazepine class, it is important to consider the potential risk of postoperative cognitive impairment. While there are few studies on the postoperative cognitive impairment induced by remimazolam, it should be advisable to continue vigilance and conduct further research.

This study also had some limitations. First, most of the included studies had possible bias and were rated as moderate in the overall assessment section, leading to some bias and possible heterogeneity and ultimately to a lower level of evidence. Second, all studies, regardless of where they were published, were conducted in China and lacked data from other countries and regions, which may result in findings that are not generalizable. Third, there was publication bias for the hypotension and recovery outcome. Improving the level of evidence for these two outcomes requires more rigorous, large-sample study support.

In conclusion, compared with propofol, remimazolam can be safely used for gastroscopic sedation and reduce the incidence of respiratory depression, hypoxemia, bradycardia, injection pain, and dizziness, and doesn't increase the incidence of nausea and vomiting, or cough. Remimazolam had a slightly longer induction time than propofol, but there was no difference in recovery or discharge time.

### Supplementary Information

**Additional file 1: Table S1.** Search strategy.

**Additional file 2: Table S2.** General information of included studies.

**Additional file 3: Figure S1.** Forest plots of Respiratory depression.

**Additional file 4: Figure S2.** Forest plots of Hypomexia.

**Additional file 5: Figure S3.** Forest plots of Bradycardia.

**Additional file 6: Figure S4.** Forest plots of Hypotension.

**Additional file 7: Figure S5.** Forest plots of Dizziness.

**Additional file 8: Figure S6.** Forest plots of Nausea or vomiting.

**Additional file 9: Figure S7.** Forest plots of Cough.

**Additional file 10: Figure S8.** Forest plots of Body movement.

**Additional file 11: Figure S9.** Forest plots of Injection pain.

**Additional file 12: Figure S10.** Forest plots of Induction time (measured in second).

**Additional file 13: Figure S11.** Forest plots of Discharge time (measured in minute).

**Additional file 14: Figure S12.** Forest plots of Recovery time (measured in second).

**Additional file 15: Figure S13.** Sensitivity analysis using one by one elimination method.

**Additional file 16: Figure S14.** Forest plots of Injection pain under sensitivity analysis.

**Additional file 17: Figure S15.** Risk of bias graph.

## Data Availability

The original contributions presented in the study are included in the article/supplementary material, further inquiries can be directed to the corresponding author.
