# Supplementary material for: The safety of remimazolam versus propofol in gastroscopic sedation: a meta-analysis
Source: BMC Anesthesiol. 2024 Jan 29;24:40. doi: 10.1186/s12871-024-02422-y (PMC10823673; doi:10.1186/s12871-024-02422-y)
Supplement: Supplementary file 1 — Additional file 1: Table S1. Search strategy. [file 12871_2024_2422_MOESM1_ESM.docx]

**Table S1. Search strategy**

| **Our search strategy in Pubmed:** | |
| --- | --- |
| (("remimazolam"[Supplementary Concept] OR "remimazolam"[All Fields] OR ((("ONO"[All Fields] AND "2745"[All Fields]) OR "ONO2745"[All Fields] OR "ONO-2745"[All Fields] OR ("CNS"[All Fields] AND "7056"[All Fields]) OR ("methyl"[All Fields] AND "3"[All Fields])) AND ("8 bromo 1 methyl 6"[All Fields] AND "2-pyridinyl"[All Fields] AND "4h imidazo"[All Fields] AND ("1"[All Fields] AND "2-a"[All Fields]) AND ("1"[All Fields] AND "4"[All Fields]) AND "benzodiazepin-4-yl"[All Fields]) AND "propanoate"[All Fields]) OR "benzodiazepines"[MeSH Terms]) AND ("propofol"[MeSH Terms] OR "propofol"[All Fields] OR "propofol s"[All Fields] OR ("mol cell ther"[Journal] OR "mol cancer ther"[Journal] OR "mct"[All Fields]) OR ("propofol"[MeSH Terms] OR "propofol"[All Fields] OR ("propofol"[All Fields] AND "lipuro"[All Fields]) OR "propofol lipuro"[All Fields]) OR ("propofol"[MeSH Terms] OR "propofol"[All Fields] OR ("ici"[All Fields] AND "35 868"[All Fields]) OR "ici 35 868"[All Fields]) OR ("propofol"[MeSH Terms] OR "propofol"[All Fields] OR ("ici"[All Fields] AND "35 868"[All Fields]) OR "ici 35 868"[All Fields]) OR ("propofol"[MeSH Terms] OR "propofol"[All Fields]) OR ("propofol"[MeSH Terms] OR "propofol"[All Fields] OR "ici 35868"[All Fields]) OR ("propofol"[MeSH Terms] OR "propofol"[All Fields] OR "ici 35868"[All Fields]) OR ("propofol"[MeSH Terms] OR "propofol"[All Fields]) OR "propofol"[MeSH Terms]) AND ("endoscopy, gastrointestinal"[MeSH Terms] OR ("endoscopy"[All Fields] AND "gastrointestinal"[All Fields]) OR "gastrointestinal endoscopy"[All Fields] OR ("gastrointestinal"[All Fields] AND "endoscopy"[All Fields]) OR ("gastroscopy"[MeSH Terms] OR "gastroscopy"[All Fields] OR "gastroscopies"[All Fields]) OR ("gastroscoped"[All Fields] OR "gastroscopes"[MeSH Terms] OR "gastroscopes"[All Fields] OR "gastroscope"[All Fields] OR "gastroscopic"[All Fields] OR "gastroscopically"[All Fields]) OR (("endoscope s"[All Fields] OR "endoscoped"[All Fields] OR "endoscopes"[MeSH Terms] OR "endoscopes"[All Fields] OR "endoscope"[All Fields] OR "endoscopical"[All Fields] OR "endoscopically"[All Fields] OR "endoscopy"[MeSH Terms] OR "endoscopy"[All Fields] OR "endoscopic"[All Fields]) AND ("stomach"[MeSH Terms] OR "stomach"[All Fields] OR "stomachs"[All Fields] OR "stomach s"[All Fields] OR "stomachal"[All Fields] OR "stomaches"[All Fields])) OR "gastroscopes"[MeSH Terms])) AND (clinicaltrial[Filter] OR randomizedcontrolledtrial[Filter]) | |
| **Our search strategy in WOS:** | |
| **#1** | TS=(remimazolam OR ONO 2745ONO2745 OR "ONO-2745" OR CNS 7056 OR methyl 38 bromo 1 methyl 6 2-pyridinyl 4h imidazo1 2-a 1 4 benzodiazepin-4-yl propanoate OR Benzodiazepines) |
| **#2** | TS=(propofol OR MCT OR Propofol-Lipuro OR ICI-35,868 OR ICI 35,868 OR ICI35,868 OR ICI-35868 OR ICI 35868 OR ICI35868) |
| **#3** | TS=(gastrointestinal endoscopy OR gastroscopy OR Gastroscopes OR (Endoscopes AND stomach)) |
| **#4** | #3 AND #2 AND #1 |
| **Our search strategy in OVID:** | |
| **1** | “randomized controlled trail”.pt. |
| **2** | ”controlled clinical trail”.pt. |
| **3** | randomized.ab. |
| **4** | placebo.ab. |
| **5** | surgery.fs |
| **6** | therapy.fs |
| **7** | “radiotherapy”.fs. |
| **8** | trail.ab |
| **9** | groups.ab. |
| **10** | exp Cohort Studies/ |
| **11** | (case adj series).ab. |
| **12** | 1 or 2 or 3 or 4 or 5 or 6 or 7 or 8 or 9 or 10 or 11 |
| **13** | Animals.ab |
| **14** | Humans.ab. |
| **15** | 13 and 14 |
| **16** | 13 not 15 |
| **17** | Remimazolam.mp.[mp=ti,ab,tx,ct,gn,sy,de,sh,th,bt,ot,nm,hw,fx,kf,ox,px,rx,ui] |
| **18** | (‘cns7056’ or ‘ono2745’ or ‘ono2745’ or ‘cns).mp. [mp=ti,ab,tx,ct,gn,sy,de,sh,th,bt,ot,nm,hw,fx,kf,ox,px,rx,ui] |
| **19** | (propofol or mct or ‘propofol lipuro’ or ‘ici-25,868’ or ‘ici35868’ or ‘ici 35868’ or ici35868.mp [mp=ti,ab,tx,ct,gn,sy,de,sh,th,bt,ot,nm,hw,fx,kf,ox,px,rx,ui] |
| **20** | exp *propofol/ |
| **21** | (‘gastroinitestinal endoscopy’ or gastroscopy or gastroscopes).mp. [mp=ti,ab,tx,ct,gn,sy,de,sh,th,bt,ot,nm,hw,fx,kf,ox,px,rx,ui] |
| **22** | 17 or 18 |
| **23** | 20 or 21 |
| **24** | 21 and 22 and 23 |
| **25** | 16 and 24 |
| **Our search strategy in Embase:** | |
| **1** | 'remimazolam'/exp OR remimazolam |
| **2** | 'cns7056' OR 'ono2745' OR 'ono 2745' OR 'cns |
| **3** | #1 OR #2 |
| **4** | propofol OR mct OR 'propofol lipuro' OR 'ici-35,868' OR 'ici 35,868' OR ici35,868 OR 'ici 35868' OR ici35868 |
| **5** | 'propofol'/exp |
| **6** | #4 OR #5 |
| **7** | 'gastrointestinal endoscopy' OR gastroscopy OR gastroscopes OR 'endoscopes near/1stomach' |
| **8** | #3 AND #6 AND #7 |
| **Our search strategy in Cochrane:** | |
| **1** | remimazolam |
| **2** | remimazolam OR ONO 2745 OR ONO2745 OR ONO-2745 OR CNS 7056 |
| **3** | Benzodiazepines |
| **4** | Propofol OR MCT OR Propofol-Lipurg OR ICI35,868 OR IC |
| **5** | propofol |
| **6** | gastrointestinal endoscopy OR gastroscopy OR gastroscope |
| **7** | gastroscope |
| **8** | #1 OR #2 OR #3 |
| **9** | #5 OR #4 |
| **10** | #6 OR #7 |
| **11** | #8 AND #9 AND #10 |
