# Supplementary material for: The safety of remimazolam versus propofol in gastroscopic sedation: a meta-analysis
Source: BMC Anesthesiol. 2024 Jan 29;24:40. doi: 10.1186/s12871-024-02422-y (PMC10823673; doi:10.1186/s12871-024-02422-y)
Supplement: Supplementary file 2 — Additional file 2: Table S2. General information of included studies. [file 12871_2024_2422_MOESM2_ESM.docx]

| **Studies** | **Patients** | **Remimazolam** | **Propofol** | **Salt Formation** | **Analgesic** | **Criteria** | **Age(year)** | **BMI**  **(kg/m^2^)** | **Gender**  **(M/F)** | **ASA**  **(I/II/III)** |
| --- | --- | --- | --- | --- | --- | --- | --- | --- | --- | --- |
| Cao YH 2022 | 148 | Induction:0.107mg/kg(30s)  Added:20mg propofol | Induction:2.0mg/kg  Added:20mg propofol | Tosilate | Sufentanil  0.15μg/kg | Induction:MOAA/S≤1  Added: MOAA/S≥2 | 48.05±6.48 | - | 87/61 | 0/122/26 |
| Guo J 2022 | 77 | Induction:0.15 mg/kg(30s)  Added:0.05 mg/kg | Induction:1.5mg/kg  Added:0.5mg/kg | Tosilate | Alfentanil  5 μg/kg | Induction:MOAA/S≤1  Added: MOAA/S≥2 | 69.76±3.98 | 23.00±3.18 | 47/30 | 14/63/0 |
| Hu BL 2022 | 346 | Induction:0.2mg/kg(60s)  Added:0.06 mg/kg | Induction:1.5mg/kg  Added:0.5mg/kg | Tosilate | Sufentanil  0.1μg/kg | Induction: MOAA/S≤3  Added: MOAA/S≥4 | 70.02±7.46 | 22.74±3.19 | 141/205 | 46/284/16 |
| Tan YJ 2022 | 66 | Induction:0.10mg /kg  Added:0.05 mg/kg | Induction:1.0~1.5mg/kg  Added:0.5mg/kg | Tosilate | Butorphanol  0.01mg/kg | Induction:MOAA/S≤1  Added: MOAA/S≥2 | 66.3±4.86 | 22.95±2.99 | 40/26 | 36/30/0 |
| Xu C 2022 | 914 | Induction:0.20mg/kg  Added:2.5mg | Induction:1.5mg/kg  Added:0.5mg/kg | Besylate | Alfentanil  10 μg/kg | Induction:patient condition  Added: patient condition | 52.63±12.90 | 23.91±3.45 | 420/494 | 0/914/0 |
| Shi WY 2022 | 161 | Induction:0.33mg/kg  Added:2.5mg | Induction:2.5mg/kg  Added:0.5mg/kg | Besylate | Alfentanil  5 μg/kg | Induction:patient condition and BIS<65  Added: patient condition | 43.73±11.24 | 22.54±1.54 | 78/83 | - |
| Ge Q 2022 | 80 | Induction:0.3mg/kg  Added:5mg | Induction:1.5mg/kg  Added:0.5mg/kg | - | none | Induction: MOAA/S≤3  Added: MOAA/S≥4 | 69.3±5.15 | 24.65±1.57 | 38/42 | 0/41/39 |
| Guo S 2022 | 100 | Induction:0.2mg/kg  Added:2.5mg | Induction:2.0mg/kg  Added:0.5mg/kg | Tosilate | Fentanyl  100μg | Induction:Loss of eyelash reflex  Added: body movement | 43.71±10.88 | 22.50±2.53 | 39/61 | 80/20/0 |
| Lu SH 2022 | 60 | Induction:0.18 mg /kg  Added:3mg | Induction:2.0mg/kg  Added:0.6mg/kg | Tosilate | dezocine | Induction: MOAA/S≤2  Added: MOAA/S≥3 | 48.55±9.48 | - | 31/29 | - |
| Teng YP 2021 | 60 | Induction:0.2mg/kg  Maintain:0.2mg/(kg·h) | Induction:1.5mg/kg  Maintain:2 mg/(kg·h) | Tosilate | Sufentanil  0.1μg/kg | Induction:Loss of eyelash reflex  Added: body movement or swallow | 71.25±5.92 | 21.95±2.40 | 26/34 | 0/33/27 |
| Zhang L 2022 | 186 | Induction:0.2mg/kg  Added:2.5mg | Induction:1.5mg/kg  Added:0.5mg/kg | Besylate | Alfentanil  7 μg | Induction: MOAA/S≤3  Added: body movement or cough | 69.00±3.76 | 23.57±3.39 | 89/97 | 0/127/59 |
| Zhang Y 2022 | 84 | Induction:0.2mg/kg  Added:2mg | Induction:2.0mg/kg  Added:20mg | Tosilate | Sufentanil  0.1μg/kg | Induction: MOAA/S≤2  Added: MOAA/S≥4 | 49.95±9.99 | - | 37/47 | 40/44/0 |
| Zhao J 2021 | 60 | Induction:0.2mg/kg  Added:2.5mg | Induction:1.5~2.0mg/kg  Added:0.5mg/kg | Tosilate | Sufentanil  0.1μg/kg | Induction:Loss of eyelash reflex  Added: patient condition | 42.50±9.75 | - | 38/22 | 33/27/0 |
| Shi CC 2021 | 60 | Induction:0.2mg/kg  Added:0.05 mg/kg | Induction:2.0mg/kg  Added:0.5 mg/kg | Tosilate | Fentanyl  1μg/kg | Induction: MOAA/S≤3  Added: patient condition | 2.56±1.14 | - | 36/24 | 47/13/0 |
| Ma GS 2022 | 80 | Induction:0.15 mg/kg  Added:0.05 mg/kg | Induction:1.5mg/kg  Added:0.5 mg/kg | - | Sufentanil  0.1μg/kg | Induction: MOAA/S≤0  Added: patient condition | 69.00±6.13 | 32.75±1.64 | 41/39 | 18/53/9 |
| Guo M 2021 | 91 | Induction:0.3mg/kg  Added:0.075 mg/kg | Induction:1.5mg/kg  Added:0.375 mg/kg | Tosilate | Remifentanil  0.25 μg/kg | Induction: MOAA/S≤3  Added: body movement | 48.36±12.66 | 24.08±2.69 | 57/34 | - |
| Kang XX 2022 | 148 | Induction:0.15 mg/kg  Added:2.5mg | Induction:1.5mg/kg  Added:0.5 mg/kg | Besylate | Alfentanil  7 μg | Induction: MOAA/S≤3  Added: MOAA/S≥4 | 51.69±11.65 | - | 73/75 | - |
| Chen ML 2022 | 80 | Induction:0.3mg/kg  Maintain:0.3~0.5mg/(kg·h) | Induction:2.0mg/kg  Maintain:3~5 mg/(kg·h) | Tosilate | Sufentanil  0.1μg/kg | Induction: MOAA/S≤0  maintain: MOAA/S≤1  Add：body movement | 48.90±8.74 | 22.77±2.45 | 42/38 | - |
| Jia T 2022 | 100 | Induction:0.3mg/kg  Added:0.075 mg/kg | Induction:1.5mg/kg  Added:0.375 mg/kg | Tosilate | Remifentanil  0.25 μg/kg | Induction: MOAA/S≤3  Added: MOAA/S≥4 | 50.99±11.25 | 22.37±2.17 | 62/38 | 54/46/0 |
| Cheng XH 2022 | 100 | Induction:0.12 mg/kg  Added:0.04 mg/kg | Induction:1.5mg/kg  Added:0.5 mg/kg | Tosilate | Butorphanol  0.5mg | Induction:MOAA/S≤2  Added: body movement | 69.90±5.56 | 23.88±2.16 | 51/49 | 0/91/9 |
| Liu HY 2022 | 100 | Induction:0.3mg/kg  Added:0.04 mg/kg | Induction:1.0~2.0mg/kg  Added:0.2~0.5 mg/kg | Tosilate | Alfentanil  7 μg | Induction:MOAA/S  Added: MOAA/S | 41.52±4.16 | 23.02±2.26 | 57/43 | - |
| Yang TS 2022 | 100 | Induction:0.55 mg/kg  Added:30~50 mg propofol | Induction:2.0mg/kg  Added:30~50 mg propofol | - | none | Induction:MOAA/S≤2  Added: body movement or cough | 51.35±10.72 | 23.25±3.01 | 48/52 | - |
| Wang YX 2022 | 100 | Induction:0.3mg/kg  Added:0.075 mg/kg | Induction:1.5mg/kg  Added:0.375 mg/kg | Tosilate | Remifentanil  0.25 μg/kg | Induction: MOAA/S≤3  Added: MOAA/S≥4 | 48.9±11.98 | 23.80±2.64 | 58/42 | - |
| Li CY 2022 | 84 | Induction:0.2mg/kg  Added:2.5 mg | Induction:1.5mg/kg  Added:0.5 mg/kg | Tosilate | Sufentani  0.01μg/kg | Induction:Loss of eyelash reflex  Added: cough | 54.66±11.2 | - | 50/34 | 47/37/0 |
| He J 2022 | 64 | Induction:0.15 mg/kg  Added:3 mg | Induction:1.5mg/kg  Added:30 mg | Tosilate | Fentanyl  50μg | Induction:MOAA/S≤1  Added: body movement or cough | 52.55±2.33 | 21.1±1.66 | 51/13 | - |
| Tang D  2023 | 192 | Induction:0.2 mg/kg  Added:2.5 mg | Induction:2.5mg/kg  Added:50 mg | Tosilate | none | Induction:MOAA/S≤3  Added: MOAA/S≥3 | 68.36±3.11 | 25.40±2.07 | 114/78 | - |

Table S2. General information of included studies.
