# Supplementary figures and images for: The safety of remimazolam versus propofol in gastroscopic sedation: a meta-analysis
Source: BMC Anesthesiol. 2024 Jan 29;24:40. doi: 10.1186/s12871-024-02422-y (PMC10823673; doi:10.1186/s12871-024-02422-y)

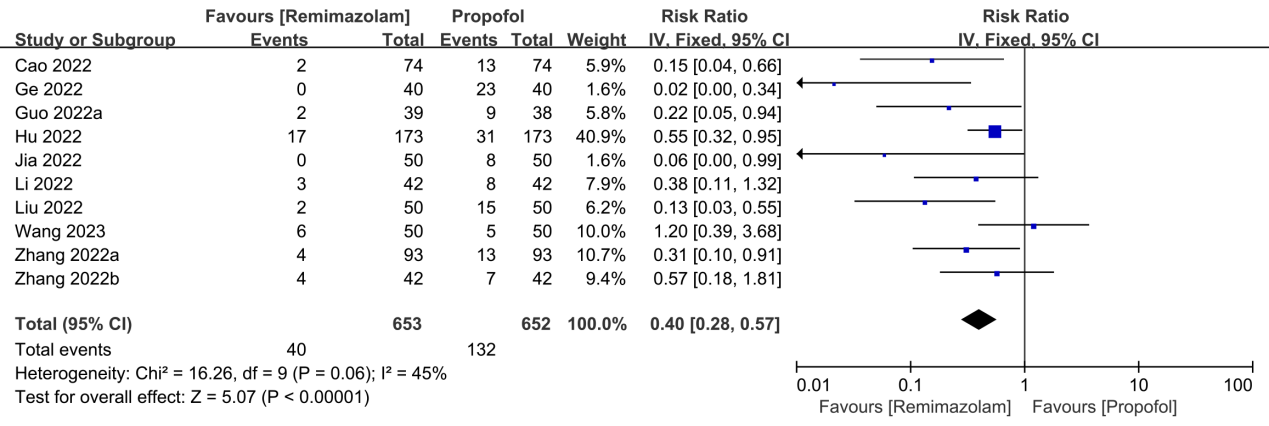


Figure S1. Forest plots of Respiratory depression

Supplement: Supplementary file 3 — Additional file 3: Figure S1. Forest plots of Respiratory depression. [file 12871_2024_2422_MOESM3_ESM.docx]

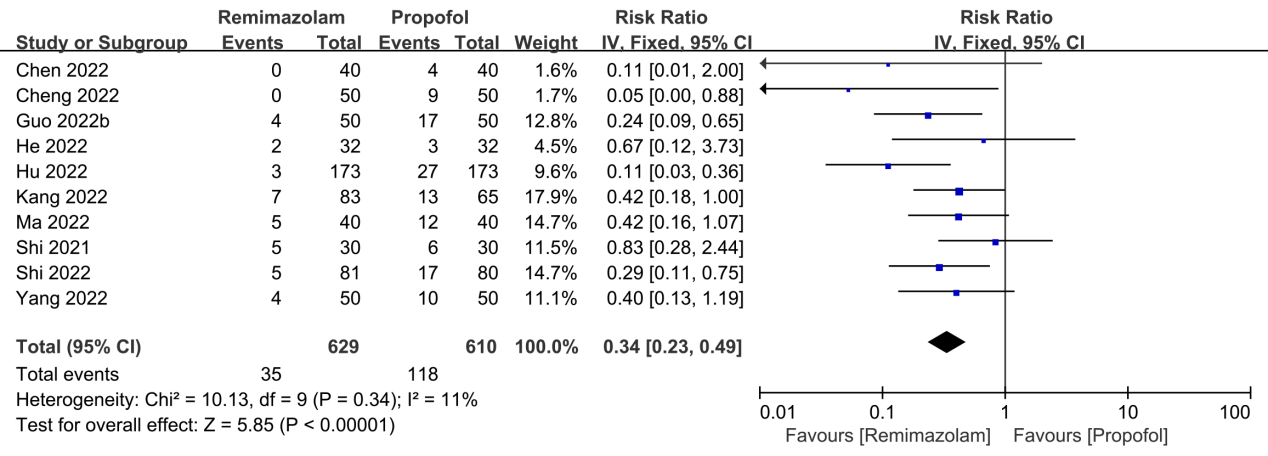


Figure S2. Forest plots of Hypomexia

Supplement: Supplementary file 4 — Additional file 4: Figure S2. Forest plots of Hypomexia. [file 12871_2024_2422_MOESM4_ESM.docx]

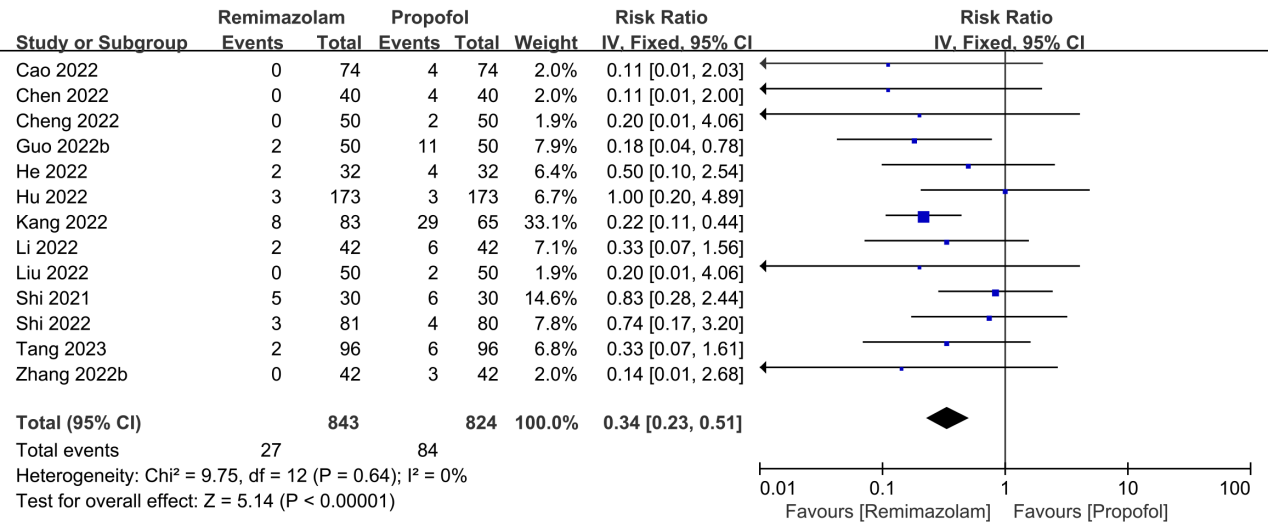


Figure S3. Forest plots of Bradycardia

Supplement: Supplementary file 5 — Additional file 5: Figure S3. Forest plots of Bradycardia. [file 12871_2024_2422_MOESM5_ESM.docx]

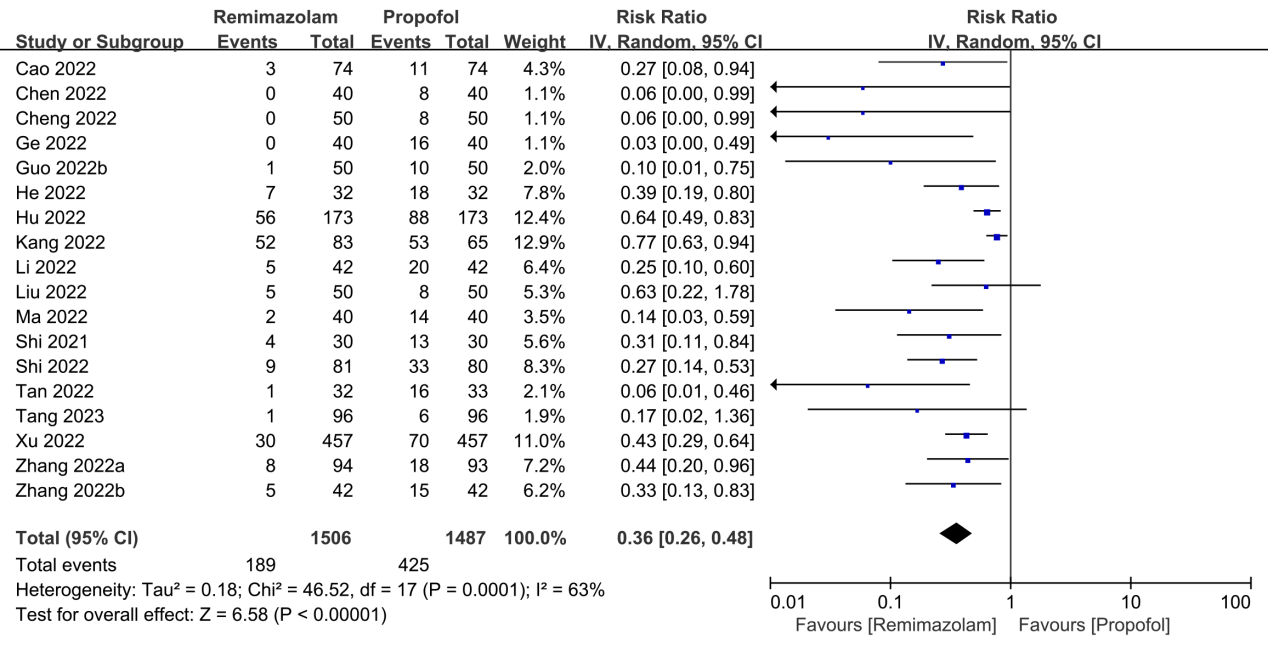


Figure S4. Forest plots of Hypotension

Supplement: Supplementary file 6 — Additional file 6: Figure S4. Forest plots of Hypotension. [file 12871_2024_2422_MOESM6_ESM.docx]

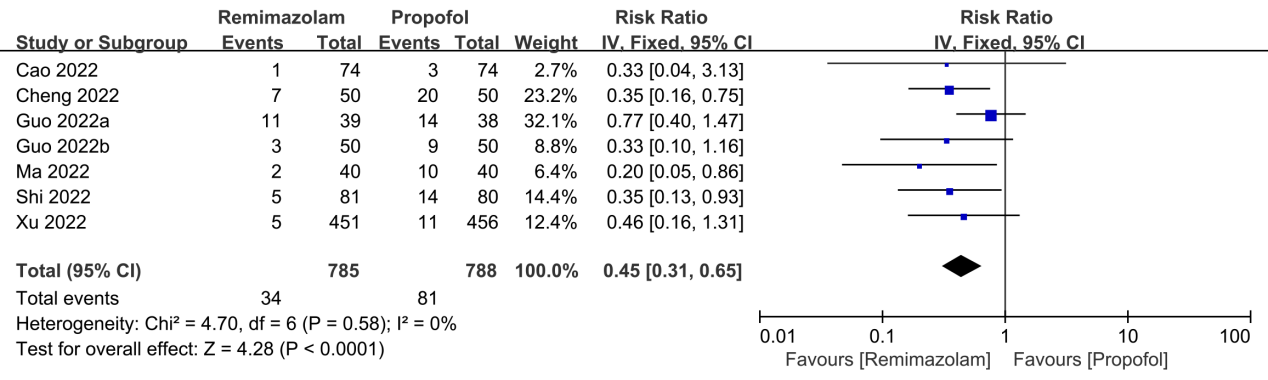


Figure S5. Forest plots of Dizziness

Supplement: Supplementary file 7 — Additional file 7: Figure S5. Forest plots of Dizziness. [file 12871_2024_2422_MOESM7_ESM.docx]

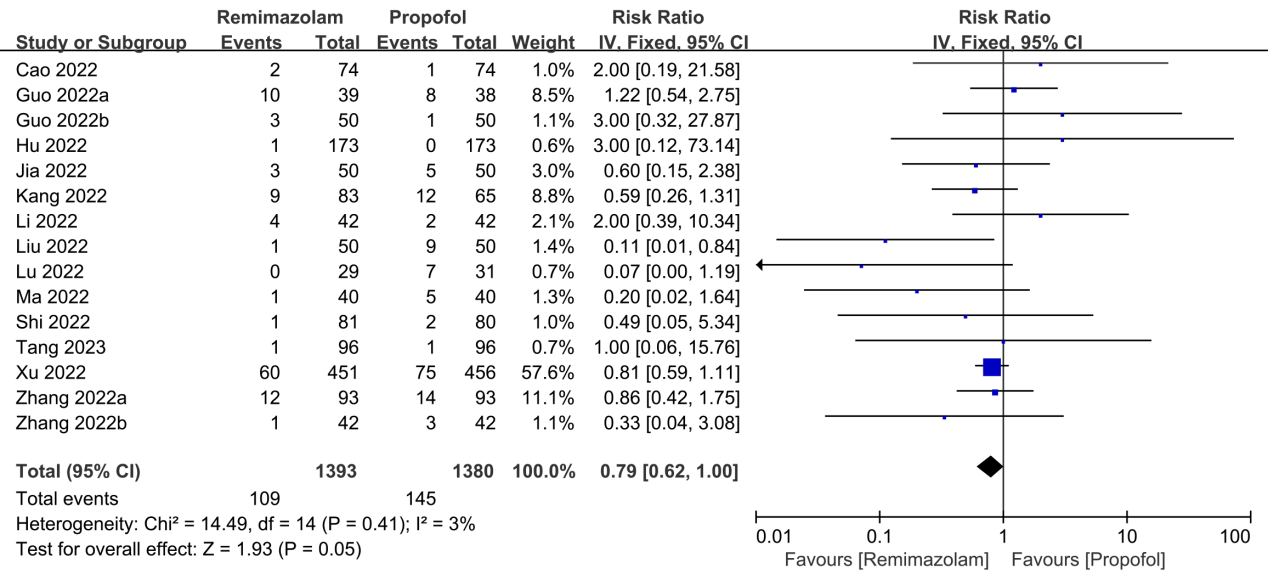


Figure S6. Forest plots of Nausea or vomiting

Supplement: Supplementary file 8 — Additional file 8: Figure S6. Forest plots of Nausea or vomiting. [file 12871_2024_2422_MOESM8_ESM.docx]

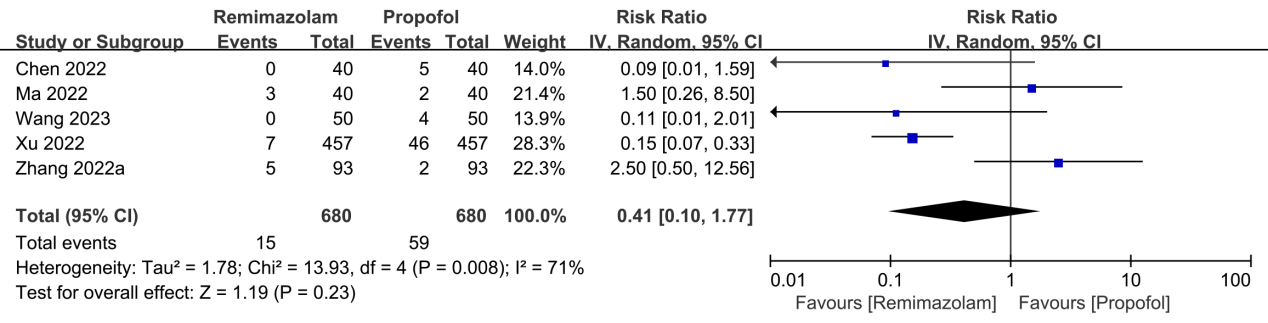


Figure S7. Forest plots of Cough

Supplement: Supplementary file 9 — Additional file 9: Figure S7. Forest plots of Cough. [file 12871_2024_2422_MOESM9_ESM.docx]

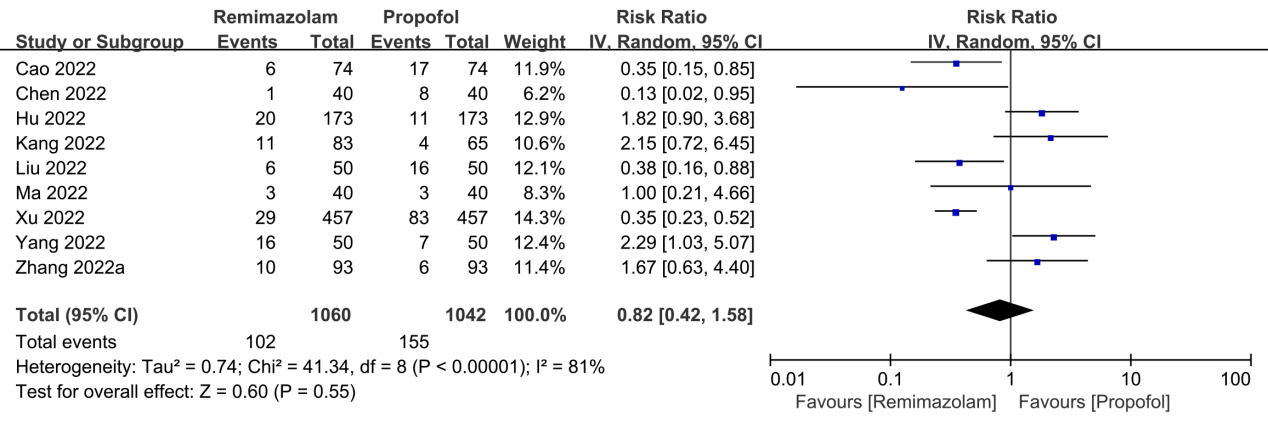


Figure S8. Forest plots of Body movement

Supplement: Supplementary file 10 — Additional file 10: Figure S8. Forest plots of Body movement. [file 12871_2024_2422_MOESM10_ESM.docx]

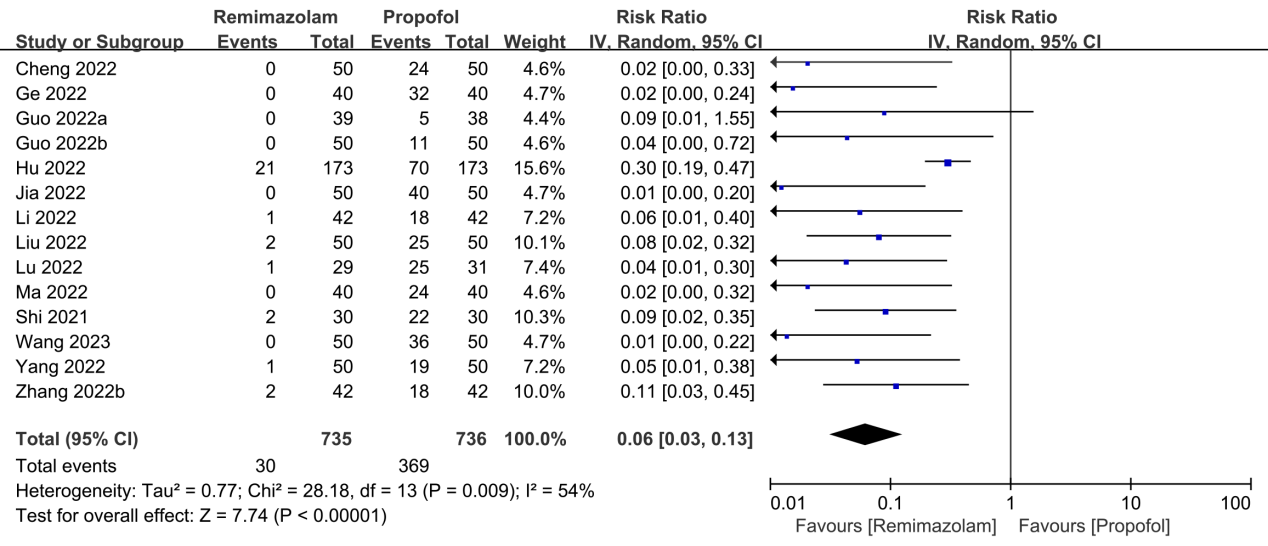


Figure S9. Forest plots of Injection pain

Supplement: Supplementary file 11 — Additional file 11: Figure S9. Forest plots of Injection pain. [file 12871_2024_2422_MOESM11_ESM.docx]

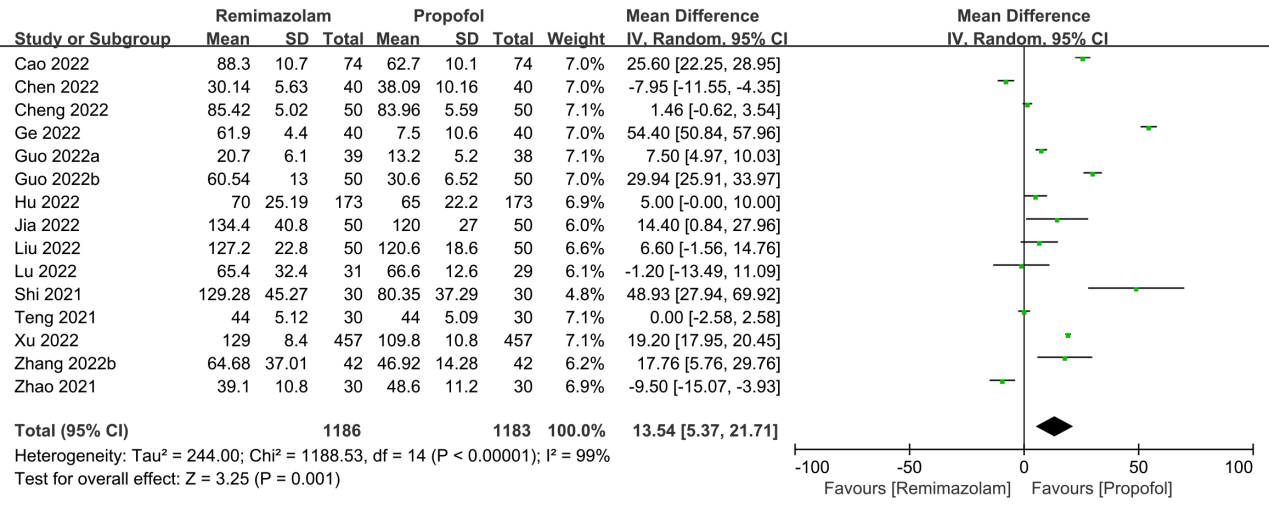


Figure S10. Forest plots of Induction time (measured in second)

Supplement: Supplementary file 12 — Additional file 12: Figure S10. Forest plots of Induction time (measured in second). [file 12871_2024_2422_MOESM12_ESM.docx]

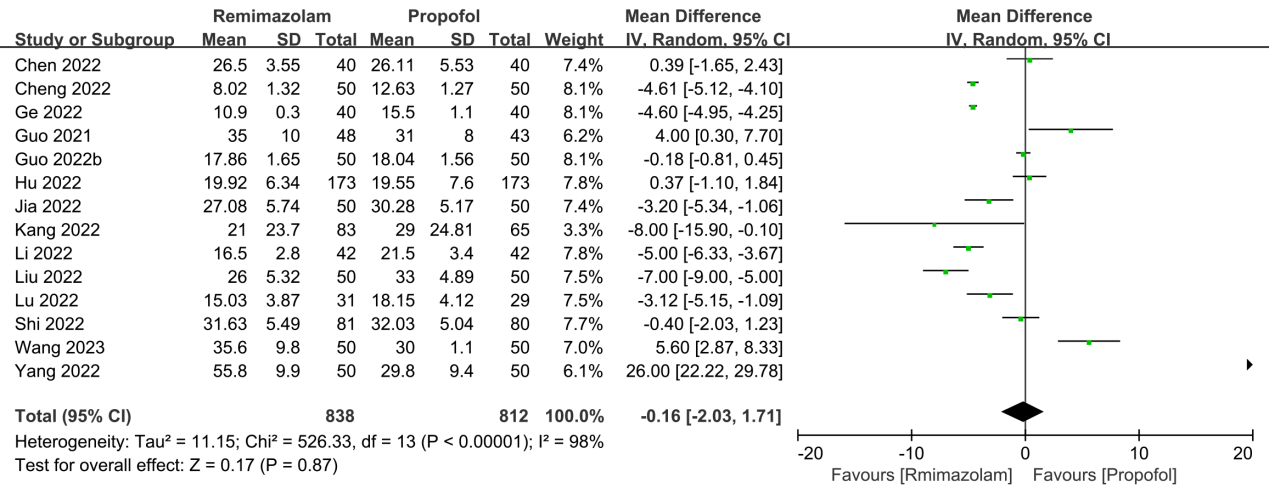


Figure S11. Forest plots of Discharge time (measured in minute)

Supplement: Supplementary file 13 — Additional file 13: Figure S11. Forest plots of Discharge time (measured in minute). [file 12871_2024_2422_MOESM13_ESM.docx]

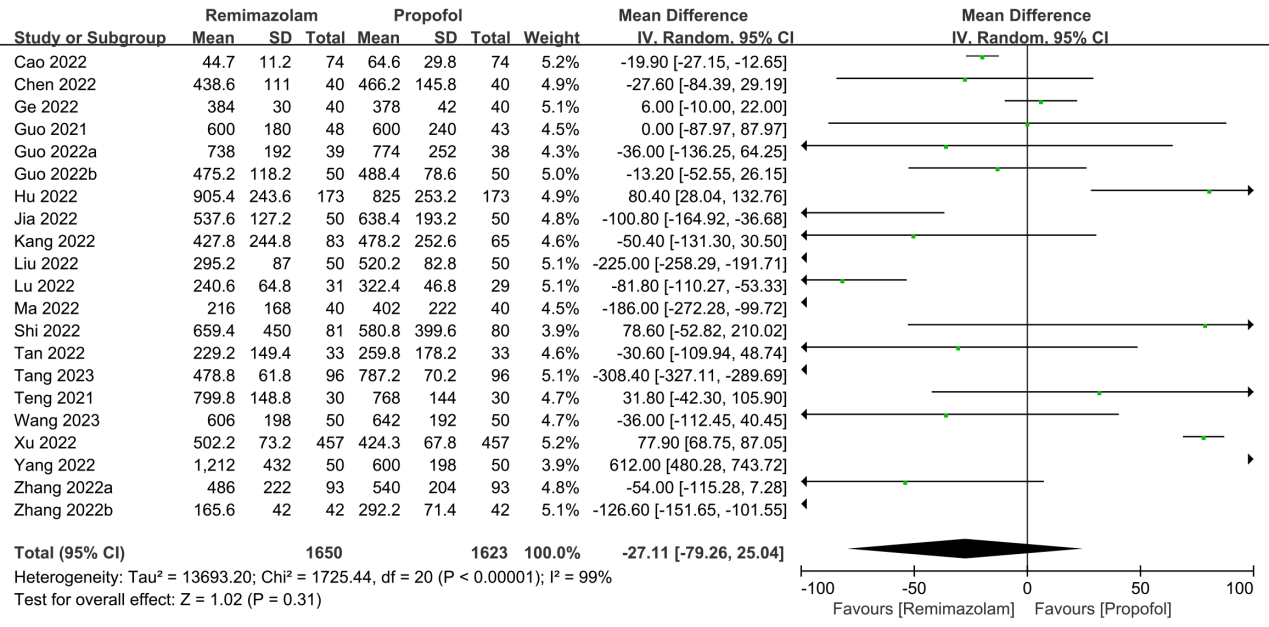


Figure S12. Forest plots of Recovery time (measured in second)

Supplement: Supplementary file 14 — Additional file 14: Figure S12. Forest plots of Recovery time (measured in second). [file 12871_2024_2422_MOESM14_ESM.docx]

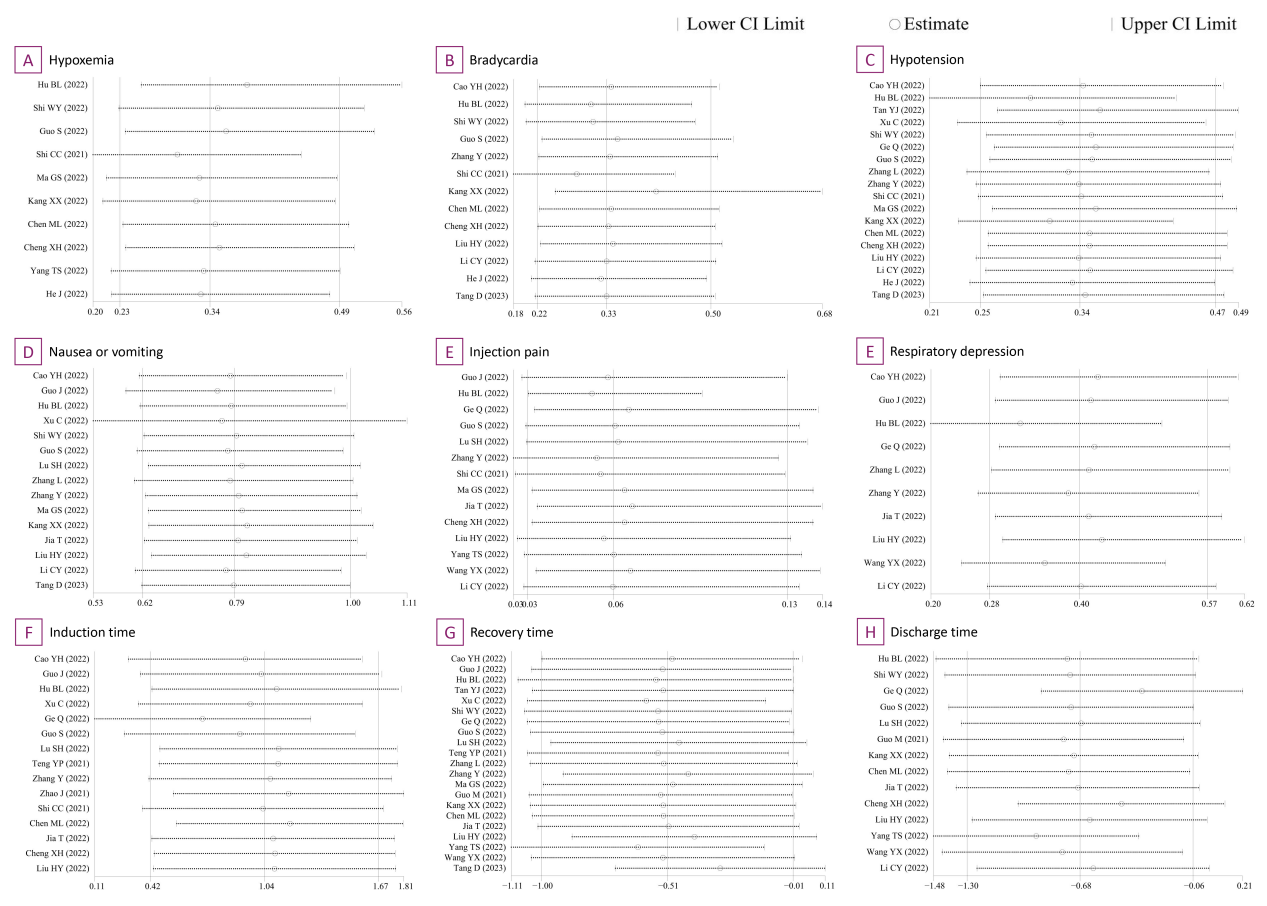


Figure S13. Sensitivity analysis using one by one elimination method

Supplement: Supplementary file 15 — Additional file 15: Figure S13. Sensitivity analysis using one by one elimination method. [file 12871_2024_2422_MOESM15_ESM.docx]

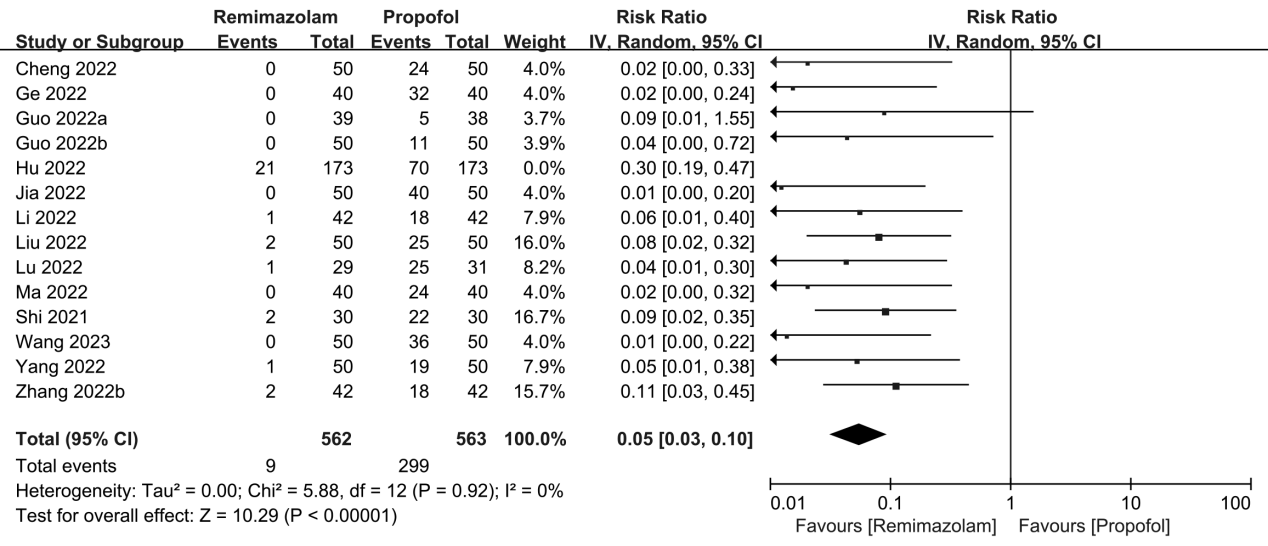


Figure S14. Forest plots of Injection pain under sensitivity analysis

Supplement: Supplementary file 16 — Additional file 16: Figure S14. Forest plots of Injection pain under sensitivity analysis. [file 12871_2024_2422_MOESM16_ESM.docx]

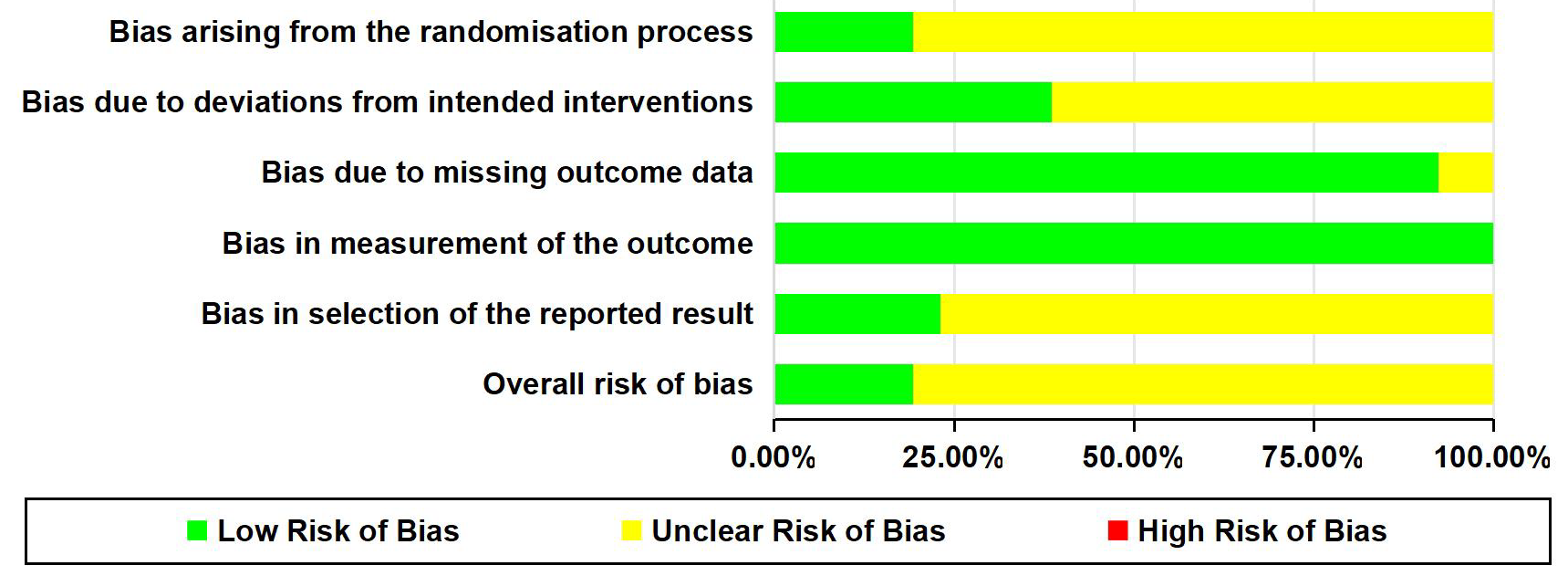


Figure S15. Risk of bias graph

Supplement: Supplementary file 17 — Additional file 17: Figure S15. Risk of bias graph. [file 12871_2024_2422_MOESM17_ESM.docx]
